# Aerated Static Pile Composting for Industrial Biowastes: From Engineering to Microbiology

**DOI:** 10.3390/bioengineering10080938

**Published:** 2023-08-07

**Authors:** Zi Xiang Keng, Jamie Jean Minn Tan, Bao Lee Phoon, Chee Chang Khoo, Ianatul Khoiroh, Siewhui Chong, Christinavimala Supramaniam, Ajit Singh, Guan-Ting Pan

**Affiliations:** 1 Department of Chemical and Environmental Engineering, University of Nottingham, Broga Road, Semenyih 43500, Selangor, Malaysia; jaron.keng@gmail.com (Z.X.K.); ianatul.khoiroh@nottingham.edu.my (I.K.); 2 School of Biosciences, University of Nottingham, Broga Road, Semenyih 43500, Selangor, Malaysia; jtjay14@gmail.com (J.J.M.T.); cheechang-96@hotmail.com (C.C.K.); 3 Nanotechnology & Catalysis Research Centre (NANOCAT), Institute for Advanced Studies, IPS Building, University of Malaya, Kuala Lumpur 50603, Malaysia; phoonpauline@um.edu.my; 4 Xodus Group, Level 1/1 William Street, Perth, WA 6000, Australia; 5 School of Science, The University of Greenwich, Chatham ME4 4TB, UK; 6 College of Science, Health, Engineering and Education, Murdoch University, 90 South Street, Murdoch, WA 6150, Australia

**Keywords:** composting, biowaste, open air, metagenome, CN ratio

## Abstract

This work demonstrated the feasibility of an industrial-scale aerated static pile composting system for treating one of the common biowastes—soybean curd residue. The mixing ratios of the feedstock were optimized to achieve a carbon–nitrogen ratio and a moisture level in the ranges of 25–35 and 60–70%, respectively. This open-air composting system required 6–7 months to obtain a mature compost. Solvita and seed germination tests further confirmed the maturity of the compost, with 25% compost extract concentration yielding the best germination index in the absence of phytotoxicity. The bacterial and fungal compositions of the compost piles were further examined with metagenomic analysis. *Thermoactinomyces* spp., *Oceanobacillus* spp., and *Kroppenstedtia* spp. were among the unique bacteria found, and *Diutina rugosa*, *Thermomyces dupontii*, and *Candida taylorii* were among the unique fungi found in the compost piles, suggesting the presence of good microorganisms for degrading the organic biowastes.

## 1. Introduction

Solid waste generation is increasing tremendously due to global population growth, urbanization, and industrialization. Biowastes are of particular interest among solid wastes as they post significant environmental issues, such as greenhouse gas emissions, water pollution, and air pollution [1]. Biowastes include animal waste, agriculture waste, garden waste, kitchen waste, and food waste [2]. In emerging economies and developing countries, these biowastes are often disposed of in open dumps or non-sanitary landfills [3,4]. The simple handling of this situation not only results in potential fire hazards, but also an uncontrolled release of pathogens and pollutants, creating several environmental and health concerns.

Soybean curd residue (SCR, also known as okara in Japanese), which is the main substrate used in this study, is a common industrial biowaste, especially in countries that favor soybean-related food products, such as Malaysia and Japan. About 14 million metric tons of SCR are disposed of annually for making soymilk and tofu worldwide [5,6]. SCR is a primary waste material of soybean products. SCR has a high moisture content (80–88%), which poses a challenge for its handling and storage, while the conventional drying methods are costly [7]. Being highly perishable, SCR is unsuitable for disposal in municipal landfills. Due to the high concentrations of organic matter and nutrients contained in SCR, composting methods are favorable to allow the SCR to be recycled and to form a product called compost for agricultural purposes, thereby creating a cradle-to-cradle production loop.

Composting is a natural aerobic process by which biodegradable materials undergo a partial mineralization and bio-transformations with the metabolism of a complex chain of diverse microbial activities using atmospheric oxygen under controlled conditions. The activities of these microorganisms are adjusted through the interplay of several key factors, such as the carbon-to-nitrogen (C/N) ratio, moisture content, aeration rate, temperature, and pH of the compost pile. Therefore, to enable microorganisms to thrive efficiently, they must be supplied with adequate nutrients, water, and oxygen, as well as an environment in which the thermophilic phase (temperature greater than 40 °C) can be sustained for the first and second months [8]. The main criteria for the control of the composting process include parameters related to two domains: (1) the external environment (temperature, moisture, agitation, aeration) and (2) the characteristics of the substrate (C/N ratio, porosity, moisture, pH, and nutrient content). C/N ratio is one very important parameter in the control of the composting process. The initial C/N ratio is key to achieving a successful composting process and the change in the C/N ratio can be used to gauge the degree of maturity of the compost. The appropriate C/N ratio of the initial materials should be 25–35 to achieve an effective composting rate; a C/N ratio below 20 is indicative of an acceptable maturity level in the final compost output, with values below 15 being preferable.

Moisture content is another critical parameter, especially for SCR, which contains up to 80–90% moisture. It directly affects almost all of the physical parameters, such as free air space, air permeability, and bulk density. According to the literature review, the optimal moisture content for microbial activity is between 40 and 60% of the compost’s weight [9]. Some research suggested a moisture level of 50–70% [10,11] while others suggested that the optimal moisture content for co-composting with high C/N ratios (>20) should be around 55–60% [10].

One of the important factors in compost application for agricultural purposes is compost stability and maturity. The application of immature compost may stunt plant growth as the active microbes in the compost compete for oxygen with plants. This phenomenon causes phytotoxicity to plants and is primarily caused by compost with an insufficient biodegradation of organic matter [12,13]. Extensive research has been conducted to analyze the composting processes to address these concerns and to develop methods to evaluate the maturity and stability of compost prior to its agricultural use [13,14]. Presently, there is no official definition or a universally accepted method of evaluating compost stability/maturity [12,15]. Organic and biological assessment is a more comprehensive way of presenting the properties of compost, especially in the compost stability and maturity index, which is often related to respirometry with methods such as the specific oxygen uptake rate, carbon dioxide release rate, Dewar self-heating test, and seed germination tests. The other related parameters are odor assessment, organic humin, humic and fluvic acid, volatile fatty acid, color, C/N ratio, and ammonium/nitrate ratio.

There are various composting techniques: in-vessel composting, Takakura composting [16], vermicomposting [17,18], using compost bins [19], aerated static pile [20], and windrow composting [21]. The aerated static pile and windrow composting techniques are well suited for treating biowaste at an industrial scale due to the scalability and relatively low-cost nature [22]. Aerated static pile composting is the selected topic in this study to suit the operating capacity of the biowaste treatment facility. Aerated static pile composting accelerates the thermophilic composting of organic waste through the pushing (positive pressure) or pulling (negative pressure) of air through the composting pile.

This paper evaluates the feasibility of an industrial scale aerated static pile composting system in Malaysia, for treating soybean curd residue. However, SCR alone is not a good composting material by itself due to its low carbon-to-nitrogen ratio, low porosity, and its acidic nature. Sawdust was used as the bulking agent in this study due to its high moisture retention, high availability, and low cost [23]. Chicken manure was added as it is favorable for the growth of most composting microorganisms [24]. The optimization of the initial feedstock mixing ratios to achieve the optimal C/N ratio and moisture level was demonstrated. The temperature profile was observed, and the compost maturity was evaluated using seed germination and Solvita^®^ compost tests. The germination index from the seed germination test measures the rate and percentage of germination. It has been widely recognized as a sensitive index [25]. The Solvita^®^ compost test is a very convenient procedure to measure the release of carbon dioxide (CO_2_) and volatile ammonia (NH_3_), which are the two most common gaseous emissions of active composts. The CO_2_ and NH_3_ emissions from active composts jointly provide critical clues to the status of the composting process, especially as it goes from “active” to “curing” and “mature” [26]. In addition, to aid further understanding of the composting process, metagenomic analysis based on culture-independent DNA sequencing was conducted to determine the microorganisms present in the compost piles.

Due to the lack of experimental investigations with a particular focus on the compost maturity and metagenomic analyses of the compost produced, this paper aims to develop a methodology to link the engineering and technical aspects of the composting process with the compost maturity and microbiological assessments, thereby providing a sound reference for future biowaste treatment improvement studies.

## 2. Materials and Methods

### 2.1. Materials

The monthly throughput of the industrial composting facility is about 400–500 tonnes, with industrial biowastes as the substrates. The built-up area of the composting site is about 1800 m^2^. Generally, a typical compost mixture consists of three components, which are the substrate, amendment agent, and bulking agent. The purpose of the amendment agent is to help to balance the C/N ratio, modify the pH value, and improve the stability. The bulking agent is a decay-resistant material providing structure and porosity to the pile. As outlined in Table 1, the substrate used in this study is soybean curd residue (SCR). Meanwhile, sawdust is the bulking agent.

### 2.2. Characterizations and Measurements

The industrial biowaste feedstocks were sent to Permulab Sdn. Bhd., Malaysia, for the analysis of moisture content, pH, organic matter, total organic carbon, total Kjeldahl nitrogen, ash content, and bulk density. A Benchtop Conductivity, TDS & Salinity Meter-IC860032 was used to measure the conductivity, salinity, and total dissolved solids. Meanwhile, the calorific value was measured using a Digital Oxygen Bomb Calorimeter XRY-1A+. All tests were duplicated, and the mean results were presented. The analysis methods and the characterization outcome of these feedstocks are presented in Table 2. The temperatures were taken at eight random positions for each pile using a temperature probe throughout the composting process.

### 2.3. Composting Method

**Figure 1:**
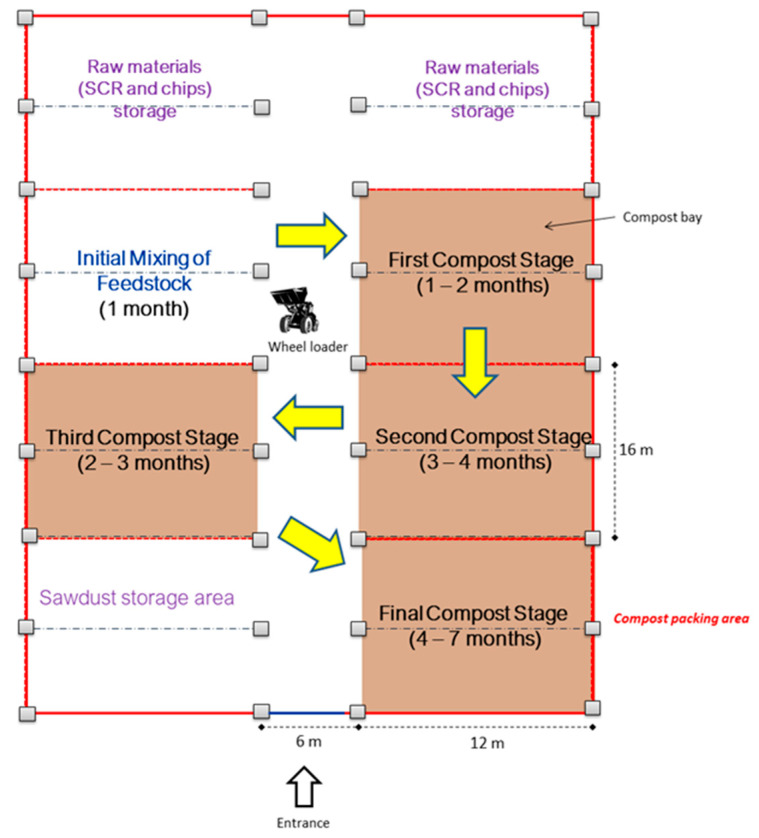
Current composting layout and process.

The design of the composting process is based on a rotational basis, in which the compost piles are mixed and moved from one bay to the next with a wheel loader. Figure 1 shows the layout of the industrial composting site. The process begins with the mixing of substrate, amendment, and bulking agents at the first bay. One month later, the first composting pile is moved to the next bay for 4 months consecutively. During these 4 months, all piles are turned every fortnight for mixing purposes. The compost was then kept at the final bay and covered with canvas for 3 months for curing and maturing purposes. Overall, the composting process takes 7 months. The existing composting process consists of four categories, which are initial mixing, rotational turning, curing, and packaging. To enhance the entire composting process, 1.7 L/t of effective microorganisms (EM) was added into the initial mix. After curing is complete, the compost should be dark brown and packed in a 50 kg gunny sacks.

The amounts of substrates added were optimized using Microsoft Excel Solver based on Equations (1) and (2), to achieve the desired C/N ratio and moisture content in the ranges of 25~35 and 60~70%, respectively [20].

(1) $$C/N=\frac{M_{1}\left[C_{1}\times \left(100-W_{1}\right)\right]+M_{2}\left[C_{2}\times \left(100-W_{2}\right)\right]+M_{3}[C_{3}\times \left(100-W_{3}\right)]+\ldots }{M_{1}\left[N_{1}\times \left(100-W_{1}\right)\right]+M_{2}\left[N_{2}\times \left(100-W_{2}\right)\right]+M_{3}\left[N_{3}\times \left(100-W_{3}\right)\right]+\ldots }$$

(2) $$W\left(\%\right)=\frac{M_{1}W_{1}+M_{2}W_{2}+M_{3}W_{3}+\ldots }{M_{1}+M_{2}+M_{3}+\ldots }$$

where C: carbon content of substrates (%); W: moisture content of substrates (%); N: nitrogen content of substrates (%); and M: weight of substrates (tonnes) with the subscript indicating the substrate number. The C and N utilized were, respectively, the total organic carbon and total Kjeldahl nitrogen in this study. The constraints in Table 3 were imposed according to the supplies of the raw materials when conducting the optimization.

### 2.4. Maturity Tests

A Solvita^®^ maturity test was conducted as one of the maturity tests. Ten sub-samples were collected from each pile and mixed thoroughly in a clean pail. Then, 1 L of the mixtures was taken for analysis. Bulky items from the mixtures were removed manually. The compost was then loaded into the Solvita jar (Woods End Laboratories Inc., Augusta, ME, USA) to the fill line (100 mL) while ensuring proper density by sharply tapping the bottom of the jar on a counter. The sample was left still for an hour. The “Carbon Dioxide” and “Ammonia” gel paddles were pushed into the compost sample in the jar. The lid of the jar was then screwed tightly and the sample kept at room temperature for 4 h. After that, the gel colors were read by comparing to the two color charts provided [27].

Another maturity analysis conducted was the seed germination assay. A total of five random samples were taken from each composting pile from the 3rd to 7th months. Then, 100 mL of distilled water was mixed with 50 g of the compost sample. The compost–water mixture was shaken for 6 h at 25 °C and centrifuged at 8000 rpm for 20 min at 20 °C [23]. The supernatant was diluted with distilled water to derive 0, 25, 50, 75, and 100% supernatant extract. Petri dishes of 10 cm in diameter were lined with cotton, and 5 mL of the samples was transferred to each of the petri dishes. Approximately 20–30 pieces of Choy sum seeds (*Brassica rapa var. Parachinensis*) were sown on each dish with two replicates per sample. After incubation at 25 °C for 72 h in the dark, the germinated seeds were counted as “G” and the root length was measured as “L”. The germination index, G_i_, was then calculated using Equation (3):

(3) $$G_{i}=\frac{G}{G_{0}}\times \frac{L}{L_{0}}\times 100$$

where G_0_ and L_0_ represent the germination percentage and root growth of the 100% distilled water as control. The global germination index, GI, is the G_i_ average of the 50% and 75% extract treatments. As a criterion, a germination index, *G_i_* above 80%, or a global germination index, GI higher than 50%, represents the non-phytotoxicity of the compost.

### 2.5. Culture-Independent DNA Sequencing

#### 2.5.1. Random Sampling

A random sampling of 50 g samples was obtained from five points from each static open-air compost pile of different maturity levels, that being compost piles of the 2nd, 4th, and 7th month of maturity. The samples were then brought back to the laboratory with a cold storage box and the five random samples from the five different points of each compost were mixed thoroughly to produce a single composite sample for each compost pile of different maturity. As a result, three composite samples, one for each compost pile of different maturity, were produced.

From each of the composite samples, two sample mark assays were prepared for further DNA extraction process as different primers; 16S and internal transcribed spacer (ITS) were used for the bacteria and fungi analysis. The steps above were repeated two months after the first sampling to produce three biological replicates (July, September, and November 2020 batches).

#### 2.5.2. DNA Extraction and Verification

A total of nine samples (three samples for each month) were stored at a temperature of 4 °C. Then, 250 mg of each of the nine samples was collected from the sample bags and DNA extraction was performed using the DNeasy PowerSoil Pro DNA Extraction kit following the manufacturer’s instructions. The quantity and quality of the extracted DNA were then determined using the Nanodrop technique. Subsequently, samples determined to contain a minimum volume of 20 ng/µL of DNA with the ratio of 260 nm/280 nm of 1.8 were selected for PCR screening using universal primers for 16S rRNA in bacteria (338F (5′-GTA CTC CTA CGG GAG GCA GCA G-3′), 533R (5′-TTA CCG CGG CTG CTG GCA C-3′)) and ITS region in fungi (ITS 1f (5′-CTT GGT CAT TTA GAG GAA GTA A-3′), ITS 2 (5′-GCT GCG TTC TTC ATC GAT GC-3′)) [28]. The PCR products were then separated by gel electrophoresis to identify DNA samples with amplified bacterial and fungal DNA fragments. That was to verify the presence of bacterial and fungal DNA within the samples before submission for sequencing. The samples that had their 16S rRNA and ITS regions successfully amplified were then sent to GeneSEQ Sdn. Bhd., Malaysia, for DNA sequencing using the Next Generation Sequencing (NGS) Illumina sequencing method. There, two sets of analyses were performed, which were bacterial composition analysis and fungal composition analysis.

#### 2.5.3. Metagenomic Analysis of Bacterial and Fungal Diversity in Compost

To sequence and analyze the bacterial composition, amplicon sequencing was performed by targeting the microbial 16S rRNA V4 gene region of extracted sample genomic DNA for amplification during PCR. The OneTaq 2X Master Mix (NEB, Ipswich, MA, USA) with the primer pair 515F-806R containing partial Illumina Nextera adapter at their 5′ ends were used for the PCR.

For the analysis of the fungal composition in the sample, the ITS1–ITS2 gene was amplified by PCR using the OneTaq 2X Master Mix with the primers ITS1f (CTTGGTCATTTAGAGGAAGTAA) and ITS2 (GCTGCGTTCTTCATCGATGC) containing partial Illumina Nextera adapter at their 5′ ends.

For both bacterial and fungal analysis, the PCR conditions were 94 °C for 30 s, followed by 35 cycles of 94 °C for 15 s, 48 °C for 15 s, and finally 68 °C for 30 s. The PCR products were then purified by immobilization of DNA fragments onto SPRI beads and used in an index PCR reaction to incorporate a dual-index barcode and Illumina adapter sequences into amplicons. The resultant index PCR products were subsequently pooled and purified before being quantified using a Denovix high-sensitivity fluorescence quantification kit. Finally, the sequencing of amplified 16S rRNA V4 amplicons was performed on an iSeq100 at 1 × 300 bp configuration.

The data analysis of the bacterial 16S Illumina sequencing began with the trimming of raw single-end demultiplexed reads using cutadapt v1.18 (San Diego, CA, USA), removing non-biological forward/reverse primer sequences at the end of each read. In addition, the data of fungal ITS sequencing generated in the form of single-end demultiplexed fastq files were trimmed using cutadapt v1.18, removing non-biological forward/reverse primer sequences at the 5′ and 3′ end of each read. The trimmed reads then acted as the input for amplicon sequence variant (ASV) generation and abundance table construction using dada2 within the QIIME2 v2020.8 pipeline.

For the analysis of the bacterial composition, the taxonomic assignment of ASVs was achieved using the QIIME2 scikit-learn naïve Bayes machine-learning classifier trained on the Genome Taxonomy Database r95. The non-mitochondrial and non-chloroplast ASVs minimally classified to the phylum level were then used to construct an ASV abundance table. The filtered abundance table, taxonomic assignment output, and sample metadata were analyzed on the MicrobiomeAnalystCA webserver to produce an overview of alpha- and beta diversity as well as the bacterial biodiversity of the samples. Similarly, for the analysis of the fungal composition, a QIIME2 scikit-learn naïve Bayes machine-learning classifier trained on the UNITE database was used for the taxonomic assignment of ASVs, and non-mitochondrial and non-chloroplast ASVs that were classified at least to the phylum level were used in the construction of an ASV abundance table. Finally, the filtered abundance table, taxonomic assignment output, and sample database underwent analysis on the MicrobiomeAnalystCA webserver.

## 3. Results and Discussion

### 3.1. Quantities and Composition of the Feedstock

**Figure 2:**
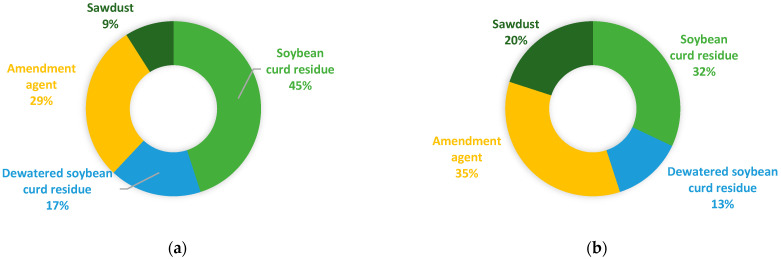
Composition of the optimized biowaste feedstock mixture (**a**) by weight in tonnes; (**b**) by volume in m^3^.

As shown in Figure 2a, the optimization method based on Equations (1) and (2) yielded a total feedstock mixture of 455 tonnes, with the main substrate, soybean curd residue, occupying 45 wt% of the feedstock mixture, followed by amendment agent (29 wt%), dewatered soybean curd residue (17 wt%), and, finally, sawdust (9 wt%). In terms of volume (Figure 2b), the amendment agent occupied the largest portion (35 vol%), followed by soybean curd residue (32 vol%), sawdust (20 vol%), and dewatered soybean curd residue (13 vol%). The resulted C/N ratio and moisture content of the feedstock mixture were 30 and 68%, respectively.

### 3.2. Temperature Profile and Maturity

**Figure 3:**
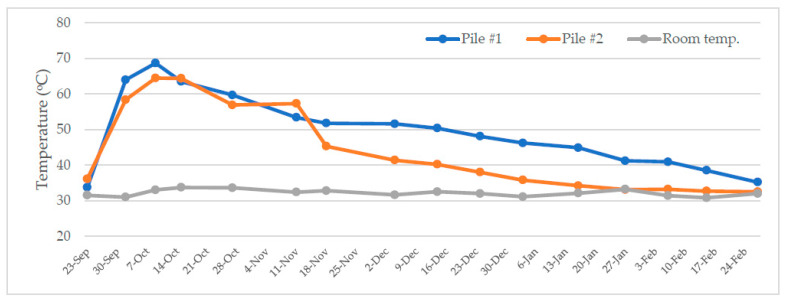
Temperature profile of the composting process.

Figure 3 shows the temperature profile of the composting process. It was found that the temperature of the composting process increased from around 35 °C on the first day to 65–70 °C after two weeks before dropping to 40–50 °C in the 3rd month. In the 5th month (24 February), the temperature gradually returned to the ambient temperature. There was about 10 °C difference during the gradual temperature drop period, which could be due to the heterogeneity of the initial feedstock for the two piles and temperature measurements at slightly different spots. Nevertheless, the temperature trends indicate a healthy and normal conventional open-air composting process.

**Figure 4:**
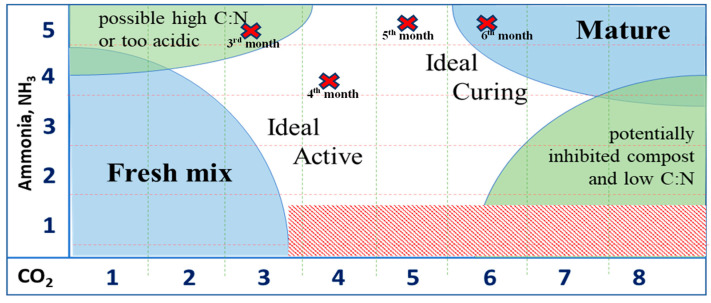
Status of composting process for the four samples of compost output.

The results of the Solvita maturity tests are shown in Table 4 and Figure 4. As shown in Table 4, the compost maturity level increased with the composting duration. Comparing Table 4 with Figure 4, a compost maturity index of 3 in the 3rd month indicates possible high C/N or too-acidic conditions due to the evolution of CO_2_ and NH_3_ from the organic matter. In the 4th month, the composting was still ideal and active, and it entered the curing stage in the 5th month. Finally, as indicated by the compost maturity index of 6, the compost started to become mature from the 6th month.

**Figure 5:**
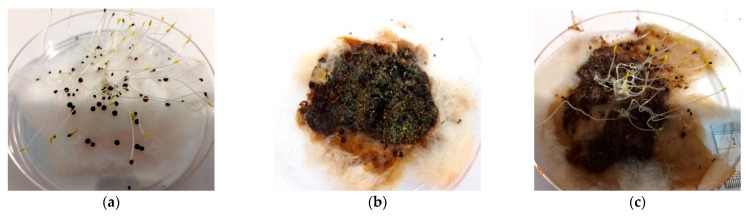

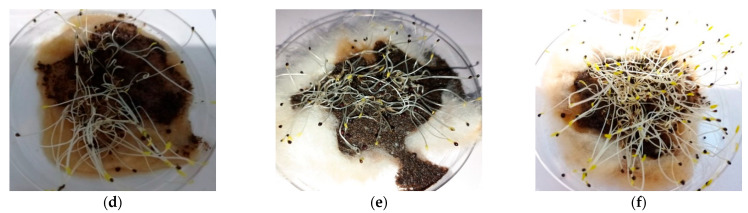
Seed germination results after 72 h of incubation at 25% extract concentration. (**a**) Control test with only distilled water; (**b**) using 3rd-month compost; (**c**) using 4th-month compost; (**d**) using 5th-month compost; (**e**) using 6th-month compost; and (**f**) using 7th-month compost.

The results of the seed germination assay are shown in Table 5. The results showed that the number of seeds germinated (and, thus, the germination index) was higher on the petri dish with 6th- and 7th-month composts compared to the rest of the compost samples. In addition, the 6th- and 7th-month composts with 25 and 50% of compost extract were found to germinate the most seeds with a higher germination index. For root length, the 6th-and 7th-month composts exhibited a relatively narrow range of variations at all concentrations, ranging from 48 to 61.5 mm. Table 5 also indicates that the 25% compost extract concentration yielded the best germination index for mature composts with the absence of phytotoxicity, i.e., the germination indexes of the 6th-and 7th-month composts at 25% extract concentration are 122 and 131, respectively (greater than 80), with the global germination indexes of 59 and 112, respectively (greater than 50). Figure 5 shows the corresponding seed germination pictures on the petri dishes using 25% extract for the composts from the 3rd to the 7th months.

### 3.3. Temperature Profile and Maturity

The bacterial and fungal profile of the composts were produced in the form of Operational Taxonomic Units (OTUs) from the metagenomic analysis performed. The amplicon sequencing variation (ASV) compositions of the three composts of different maturity were then compared with each other in a Venn diagram and the ASVs unique to and shared between each compost pile were determined. Venn diagrams of the bacterial and fungal ASVs in compost samples from the 2nd, 4th, and 7th months were generated using Venny 2.1.

**Figure 6:**
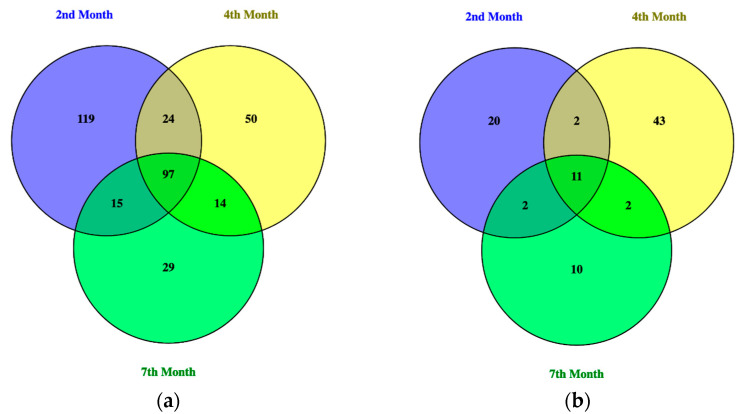
Venn diagrams of the (**a**) bacterial and (**b**) fungal ASVs found in compost piles of 2-, 4-, and 7-month maturity.

Through this visual representation, as shown in Figure 6, overlaps in bacterial and fungal compositions were found between all three compost piles of differing maturity. For the bacterial profile, 97 ASVs were shared amongst all three, 24 ASVs between the 2nd month and the 4th month, 14 ASVs between the 4th month and the 7th month, and 15 ASVs between the 2nd month and the 7th month. Some microbes were also found to be unique to each compost pile of different maturity, with 119 ASVs unique to the compost pile of 2nd month maturity, 50 ASVs unique to the 4th month, and 29 ASVs unique to the 7th month.

For the fungal profile, two ASVs were found to be shared between the 2nd and 4th month, 4th and 7th month, and 2nd and 7th month composts, while 11 common ASVs were shared between all three composts of different maturity. It was also found that the 2nd month had 20 unique ASVs, the 4th month had 43 unique ASVs, and the 7th month had 10 unique ASVs.

Table 6 denotes the top-five bacteria and fungi of each month or shared months.

**Figure 7:**
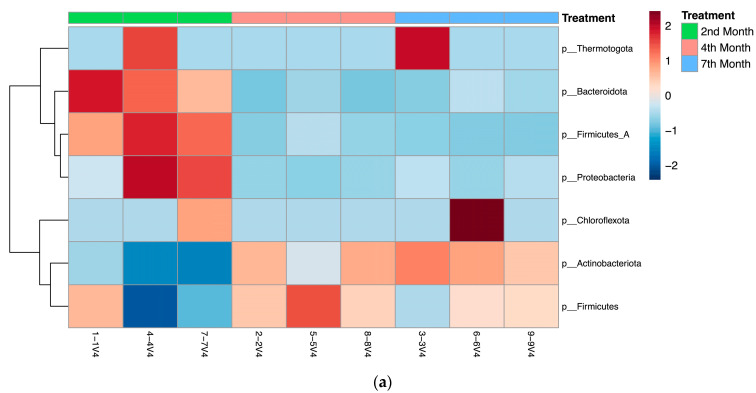

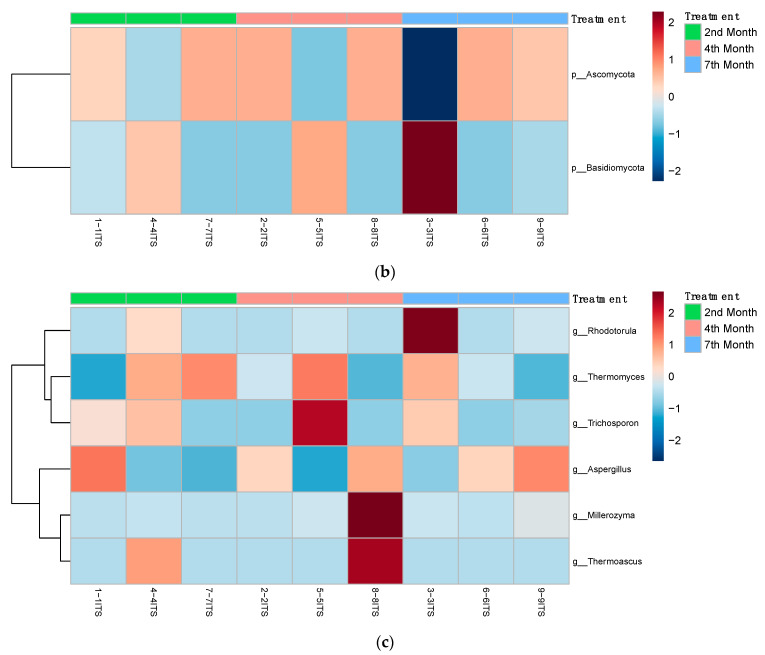
Heatmaps depicting the abundance and clustering of (**a**) bacterial phyla, (**b**) fungi at the phylum level, and (**c**) fungi at the genus level in 2nd-, 4th-, and 7th-month compost samples.

Figure 7 shows further analysis of the abundance and clustering of bacteria phyla, fungi at the phylum level, and fungi at the genus level in each sample with heatmaps generated using the MicrobiomeAnalyst webserver.

### 3.4. Further Discussions and Recommendations

As shown in Figure 7a, from the bacterial metagenomic analysis of the 2nd-, 4th-, and 7th-month composts, bacterial ASVs belonging to the *Thermotogota*, *Bacteroidota* (i.e., *Bacteroidetes*), *Firmicutes*, *Protobacteria*, *Chloroflexota*, *Actinobacteria* phyla were found to be abundant at different degrees throughout the maturing process. Zhong, et al. [29] and Partanen, et al. [30] found similar bacterial phyla abundances, but the former detected a significant abundance of *Planctomycetes* instead of *Thermotogota*, while the latter identified sequences belonging to the *Deinococcus-Thermus* phylum and did not find any belonging to the *Chlorofexota* or *Thermotogota* phyla. These differences in the microbial abundances of composts can be attributed to the different raw materials and composting process used in these studies, which can change the microbial composition within the compost significantly. *Actinobacteria* and *Bacteroidetes* are among the bacterial phyla reported to play a significant role during the maturation process [31], while *Firmicutes* is a major phylum during the thermophilic stage of composting, having a tolerance for unfavorable conditions and an ability to metabolize carbohydrates effectively [32].

As shown in Table 6, among the notable unique bacterial ASVs are ASVs for a *Thermoactinomyces* spp. (*T. vulgaris*), an *Oceanobacillus* spp. (*O. caeni*) and *Kroppenstedtia* spp. (*K. eburnea*) unique to the 2nd-, 4th-, and 7th-month composts, respectively. *Actinobacteria* and *Thermoactinomyces* spp. are indicative of a well-aerated composting system with *Thermoactinomyces* spp. being thermophilic and thermotolerant [30]. *T. vulgaris* is a bacterium that is commonly found on moldy hay [33], which aligns with the detection of its ASV in the younger, 2nd-month compost, as it may have originated from the substrates used. *Oceanobacillus* spp. are tolerant to high temperatures and salinity stress—being spore-forming and moderately halophilic—and its catalytic and oxidizing abilities are immensely helpful for organic composting [34,35]. *Kroppenstedtia* spp., similar to *Thermoactinomyces* spp., are part of the *Thermoactinomycetoceae* family and are aerobic bacteria with thermotolerant growth. von Jan, et al. [36] noted a positive urease reaction by *K. eburnea*. This suggests that *K. eburnea* may play a role in urea breakdown during composting.

As shown in Figure 7b,c, the analysis of the fungal composition of composts at the 2nd, 4th, and 7th months of maturity showed varied abundances of fungi from the *Ascomycota* and *Basidiomycota* phyla throughout the different composts with fungi from the genus *Rhodotorula*, *Thermomyces*, *Trichosporon*, *Aspergillus*, *Millerozyma*, and *Thermoascus*. Langarica-Fuentes, et al. [37] and Meng, Yang, Men, Bello, Xu, Xu, Deng, Jiang, Sheng and Wu [32] found similar abundances at the phylum level, with *Ascomycota* being the most abundant, followed by *Basidiomycota*. However, at the genus level, the genera noted in this study are different to those studies [32,37], though overlap exists. This can be explained by the differences in process operations, initial feedstock, and composting conditions involved. The *Aspergillus* genus includes saprophytic and some thermotolerant fungi that are commonly found in dead plant material and soil, and is used as an indicator group of the initial phase of composting [32,37], while more thermophilic and thermotolerant fungi from the *Thermomyces* genus are able to produce thermostable or thermoresistant enzymes for composting [38].

As shown in Table 6, the ASVs for *Diutina rugosa* (also known as *Candida rugosa*) unique to the 2nd-month compost and *Thermomyces dupontii* and *Candida taylorii* unique to the 4th-month compost were some of the notable fungi found through analysis. As found in the 2nd-month compost, Langarica-Fuentes, Zafar, Heyworth, Brown, Fox and Robson [37] similarly detected *Diutina rugosa* in the early stages of composting. Sun, et al. [39] isolated and identified *Diutina rugosa* as a yeast strain with efficient phosphorus removal capabilities for wastewater treatment. The fungus was able to provide high phosphorus content as a fertilizer when yeast sludge was processed. Therefore, in composting, this fungus may contribute to the phosphorus content of the end compost. *Thermomyces dupontii* is a key species during the thermophilic stage of compost maturation, capable of producing xylanase, phytase, and chitinase, thus allowing for the degradation of woody substrates [38]. *Candida taylorii*, on the other hand, is a fungus more commonly found in marine habitats, but is capable of fermenting several kinds of carbohydrate substrates, contributing to the composting of glucose and carbohydrate-rich feedstock [40].

As shown in the temperature profile (Figure 3) and the maturity tests (Table 5 and Table 6), the open-air composting of biowastes with an optimized mixing ratio of soybean curd residue (as-is and dewatered), amendment agent, and sawdust requires a duration of 6–7 months to achieve maturity and non-phytotoxicity. Using compost lesser than 6 months will induce phytotoxicity, which then affects crop production. The end compost produced was found to be rather wet (having a moisture level of 45%) with a low pH of 4.67, a high total organic matter close to 90%, a total organic carbon of 52% and a C/N ratio of 17. In terms of nutrient content, the total nitrogen, phosphorus, and potassium of the end compost were 3, 1.43, and 2%, respectively.

The composting process can be shortened should the pile turning be conducted with an optimized frequency (instead of once per fortnight) [41] at the expense of higher manpower and advanced machinery. In-vessel composting [42,43] could be a promising alternative to open-air composting due to its closed system where feeding, temperature, and mixing can be monitored and controlled. The integration of intelligent elements into the composting process [44,45] would be a future trend for process improvement, especially in terms of composting duration and compost quality. Economic, societal, and environmental impact analyses such as lifecycle assessment and cleaner production should be other considered aspects in future studies to achieve a circular economy for biowaste management and treatment.

## 4. Conclusions

An industrial scale biowaste open-air static composting system for the treatment of soybean curd residue was studied. The total feedstock treated was 455 tonnes, of which the main substrate was soybean curd residue, accounting for 45 wt% of the raw material mixture. As indicated by the Solvita maturity and seed germination tests, the open-air composting process took 6 to 7 months to obtain a mature compost. Metagenomic analysis was conducted to determine the common and unique bacteria and fungi in the composts of different months. Despite its many advantages, such as less turning frequency and being less labor intensive, the design/arrangement of the aeration rate plays a critical role. Insufficient aeration rates will cause the pile temperature to increase and too much aeration will result in a slower composting rate. Therefore, this creates a tradeoff that needs to be accounted for during the design and testing of the system.

## Figures and Tables

**Table 1:** Substrate, amendment agent, and bulking agent used in this study.

Component of Compost Mixture	Organic Material
Substrate	Soybean curd residue (SCR)
Amendment agent	Chicken manure and others [dataset]
Bulking agent	Sawdust

**Table 2:** Characterization results of the industrial biowaste feedstocks used.

Parameters	Method Reference	Soybean Curd Residue	Dewatered Soybean Curd Residue	Sawdust
Moisture Content (%)	AOAC931.04	89	82	27
pH at 25 °C	AOAC945.10	4.15	4.18	7.15
Organic Matter (%)	MS 417: Part 8: 1997	95	96	98
Total Organic Carbon (%)	MS 417: Part 8: 1997	56	56	57
Total Kjeldahl Nitrogen (%)	MS 417: Part 3: 1994 & AOAC991.20	4.1	3.6	0.4
Ash Content (%)	USP 24NF19	3.9	7.0	2.2
C/N Ratio (TOC/TKN)	Calculation	14.3	16.1	153.2

**Table 3:** Constraints on the amounts of substrates.

Substrate/Agent	Constraint
SCR	100–300 tonnes/cycle
Dewatered SCR	50–150 tonnes/cycle
Amendment agent	100–200 tonnes/cycle
Sawdust	40–120 tonnes/cycle
Total weight	500 tonnes
Total volume	700 m^3^

**Table 4:** Solvita maturity test outcome for the composts in the 3rd, 4th, 5th, and 6th months.

Sample	CO_2_ Result	NH_3_ Result	Compost Maturity Index
3rd-month compost	3	5	3
4th-month compost	4	4	4
5th-month compost	5	5	5
6th-month compost	6	5	6

**Table 5:** Seed germination assay outcome for the composts in the 3rd, 4th, 5th, 6th, and 7th months.

Composting Duration	Sample ID	Percentage of Compost Extract	Average Number of Seeds Germinated	Average Root Length (mm)	Germination Index (G_i_)	Global Germination Index (GI)
3 months	1.1	0	54.5	53	100	0
	1.2	25	0	0	0	
	1.3	50	0	0	0	
	1.4	75	0	0	0	
	1.5	100	0	0	0	
4 months	2.1	0	54	51	100	1.46
	2.2	25	14	11.5	5.85	
	2.3	50	11.5	7	2.92	
	2.4	75	0	0	0	
	2.5	100	0	0	0	
5 months	3.1	0	51.5	50	100	10.00
	3.2	25	35.5	47.5	65.49	
	3.3	50	10	51.5	20.00	
	3.4	75	0	0	0	
	3.5	100	0	0	0	
6 months	4.1	0	47	48	100	58.94
	4.2	25	53	52	122.16	
	4.3	50	29	52	66.84	
	4.4	75	23.5	49	51.04	
	4.5	100	7.5	51.5	17.12	
7 months	5.1	0	47.5	48	100	111.97
	5.2	25	48.5	61.5	130.82	
	5.3	50	52.5	57	131.25	
	5.4	75	39.5	53.5	92.69	
	5.5	100	20	51.5	45.18	

**Table 6:** Common microbes between the composts in the 2nd, 4th, and 7th months of maturity.

Month(s)	Bacteria	Fungi
**Common microbes**		
**2nd, 4th, and 7th months**	*s__Oceanobacillus_caeni*, *g(U)_Acetobacter*, *g(U)_Corynebacterium*, *g(U)_Corynebacterium*, *g(U)_Acetobacter*	*g(U)_Aspergillus*, *s__Thermomyces_dupontii*, *g(U)_Aspergillus*, *g(U)_Aspergillus*, *s__Thermomyces_dupontii*
**2nd and 4th months**	*f(U)_Thermoactinomycetaceae*, *s__Bacillus_O_smithii*, *s__Chishuiella_changwenlii*, *c(U)_Bacilli*, *f(U)_Streptosporangiaceae*	*s__Thermoascus_crustaceus*, *s__Thermomyces_stellatus*
**4th and 7th months**	*g(U)_Corynebacterium*, *g(U)_Corynebacterium*, *s__Corynebacterium_phoceense*, *g(U)_Corynebacterium*, *g(U)_Staphylococcus*	*g(U)_Thermomyces*, *g(U)_Aspergillus*
**2nd and 7th months**	*g(U)_Limosilactobacillus*, *s__Myroides_sp004151275*, *s__Clostridium_V_ultunense*, *g(U)_Lactobacillus*, *g(U)_Sphingobacterium*	*g(U)_Aspergillus*, *s__Rhodotorula_toruloides*
**Unique microbes**		
**2nd month**	*g(U)_Acetobacter*, *g(U)_Acetobacter*, *g(U)_Nocardiopsis*, *s__Thermoactinomyces_vulgaris*, *g(U)_Acinetobacter*	*s__Diutina_rugosa*, *f(U)_Saccharomycetales_fam_Incertae_sedis*, *s__Diutina_rugosa*, *p(U)_Ascomycota*, *f(U)_Saccharomycetales_fam_Incertae_sedis*
**4th month**	*f(U)_Thermoactinomycetaceae*, *s__Oceanobacillus_caeni*, *g(U)_Saccharopolyspora*, *s__Lentibacillus_sp902806455*, *f(U)_Amphibacillaceae*	*s__Thermomyces_dupontii*, *s__Thermoascus_crustaceus*, *s__Thermomyces_dupontii*, *s__Candida_taylorii*, *g(U)_Aspergillus*
**7th month**	*s__Kroppenstedtia_eburnea*, *g(U)_Corynebacterium*, *o(U)_Bacillales_B*, *g(U)_Corynebacterium*, *g(U)_Gordonia*	*s__Thermomyces_dupontii*, *g(U)_Melanocarpus*, *g(U)_Polypaecilum*, *s__Aspergillus_heterocaryoticus*, *g(U)_Sterigmatomyces*

## Data Availability

3rd Party Data. Restrictions apply to the availability of these data. Data was obtained from University of Nottingham Malaysia and are available [from the authors/at URL] with the permission of University of Nottingham Malaysia.
